# Heparan sulfate promotes differentiation of white adipocytes to maintain insulin sensitivity and glucose homeostasis

**DOI:** 10.1016/j.jbc.2021.101006

**Published:** 2021-07-24

**Authors:** Takuro Matsuzawa, Masanobu Morita, Ai Shimane, Rina Otsuka, Yu Mei, Fumitoshi Irie, Yu Yamaguchi, Kazuhiko Yanai, Takeo Yoshikawa

**Affiliations:** 1 Department of Pharmacology, Tohoku University Graduate School of Medicine, Sendai, Japan; 2 Department of Environmental Medicine and Molecular Toxicology, Tohoku University Graduate School of Medicine, Sendai, Japan; 3 Human Genetics Program, Sanford Burnham Prebys Medical Discovery Institute, La Jolla, California, USA

**Keywords:** heparan sulfate, adipocyte, adipogenesis, insulin resistance, diabetes, bone morphogenetic protein, fibroblast growth factor, BMP4, bone morphogenetic protein 4, DMEM, Dulbecco's modified Eagle's medium, Fabp4, fatty acid–binding protein 4, FGF1, fibroblast growth factor 1, HFD, high-fat diet, HS, heparan sulfate, HSBP, HS-binding protein, Irs, insulin receptor substrate, ITT, insulin tolerance test, LD, lipid droplet, ND, normal diet, NIH, National Institutes of Health, PPARγ, peroxisome proliferator–activated receptor gamma, TGF-β, transforming growth factor β, vWAT, visceral WAT, WAT, white adipose tissue

## Abstract

Heparan sulfate (HS), a highly sulfated linear polysaccharide, is involved in diverse biological functions in various tissues. Although previous studies have suggested a possible contribution of HS to the differentiation of white adipocytes, there has been no direct evidence supporting this. Here, we inhibited the synthesis of HS chains in 3T3-L1 cells using CRISPR–Cas9 technology, resulting in impaired differentiation of adipocytes with attenuated bone morphogenetic protein 4 (BMP4)–fibroblast growth factor 1 (FGF1) signaling pathways. HS reduction resulted in reduced glucose uptake and decreased insulin-dependent intracellular signaling. We then made heterozygous mutant mice for the *Ext1* gene, which encodes an enzyme essential for the HS biosynthesis, specifically in the visceral white adipose tissue (*Fabp4-Cre*^*+*^::*Ext1*^*flox/WT*^ mice, hereafter called *Ext1*^*Δ/WT*^) to confirm the importance of HS *in vivo*. The expression levels of transcription factors that control adipocyte differentiation, such as peroxisome proliferator–activated receptor gamma, were reduced in *Ext1*^*Δ/WT*^ adipocytes, which contained smaller, unilocular lipid droplets, reduced levels of enzymes involved in lipid synthesis, and altered expression of BMP4–FGF1 signaling molecules. Furthermore, we examined the impact of HS reduction in visceral white adipose tissue on systemic glucose homeostasis. We observed that *Ext1*^*Δ/WT*^ mice showed glucose intolerance because of insulin resistance. Our results demonstrate that HS plays a crucial role in the differentiation of white adipocytes through BMP4–FGF1 signaling pathways, thereby contributing to insulin sensitivity and glucose homeostasis.

Heparan sulfate (HS) is a linear chain of repeated disaccharide subunits that are highly sulfated on their carbohydrate residues (1). HS covalently attaches to various core proteins and forms HS proteoglycans, which are abundantly distributed on cell surfaces and in the extracellular matrix. Biosynthesis of HS chains is regulated by diverse enzymes involved in chain polymerization and modification. Chain elongation is predominantly catalyzed by an enzymatic complex of Ext1 and Ext2, which sequentially adds 50 to 250 disaccharide units of *N*-acetylglucosamine and glucuronic acid. Various sulfotransferases promote the sulfation of these HS chains, providing them with a high negative charge density. This promotes the interaction of HS with positively charged biomolecules (HS-binding protein [HSBP]), such as growth factors and cytokines, at cell surfaces. The role of HS as a coreceptor of various signaling molecules enhances ligand–receptor encounters and results in the augmentation of intracellular signaling (2). In particular, the essential role of HS in the binding of fibroblast growth factors (FGFs) to their receptors (FGF receptors) is well documented (3).

Previous studies have revealed that HS is involved in diverse physiological functions, such as developmental processes (4), synaptic organization (5), axonal guidance (6), and angiogenesis (7). Recent reports have shown that HS is associated with various pathological conditions, including autism (8) and osteochondroma (9), attracting attention to HS as a therapeutic target in various disorders. Indeed, palovarotene, a retinoic acid receptor γ selective agonist, has been proven to possess therapeutic potential in a mouse model of osteochondroma caused by HS loss (10) and is currently under clinical trial for treatment of human osteochondroma. In addition, genome-wide association studies have indicated a risk variant of an HS synthesis gene in type 2 diabetes (11, 12, 13). Our previous studies have demonstrated that HS plays important roles in the differentiation and proliferation of pancreatic β-cells and contributes to normal insulin secretion in mice (14). A small clinical study also indicated that loss-of-function mutations in *EXT1* or *EXT2* impaired insulin secretion (15). Although these studies emphasize the involvement of HS in insulin secretion, its involvement in insulin sensitivity is poorly understood.

White adipose tissue (WAT) is one of the most important tissues that determine insulin sensitivity and control glucose homeostasis. White adipocytes can store excess energy as triglycerides in a unilocular lipid droplet (LD), protecting other organs from lipotoxicity. They also act as endocrine cells, releasing various adipokines to control systemic energy homeostasis (16). Differentiation of preadipocytes to mature white adipocytes dramatically potentiates their functions in glucose uptake, energy storage, and adipokine secretion, leading to the improvement of systemic glucose homeostasis (17). Previous studies have shown a prominent increase in HS during adipocyte differentiation and the involvement of several HSBPs in the differentiation process (18), suggesting that HS in white adipocytes might modulate several signaling pathways, inducing adipogenic differentiation and leading to normal insulin sensitivity and glucose homeostasis. However, the direct involvement of HS in adipocyte function remains to be elucidated.

In the present study, we inhibited the synthesis of HS chains in 3T3-L1 adipocytes, using CRISPR–Cas9 technology, to analyze the importance of HS in adipocyte differentiation and insulin-dependent glucose uptake. Then, we specifically deleted HS in mouse WAT, using the Cre-loxP system, to examine the role of HS *in vivo*. Our results clearly demonstrate that HS promotes the differentiation of adipocytes and contributes to normal glucose homeostasis.

## Results

### HS deletion in 3T3-L1 adipocytes impaired their differentiation

**Figure 1:**
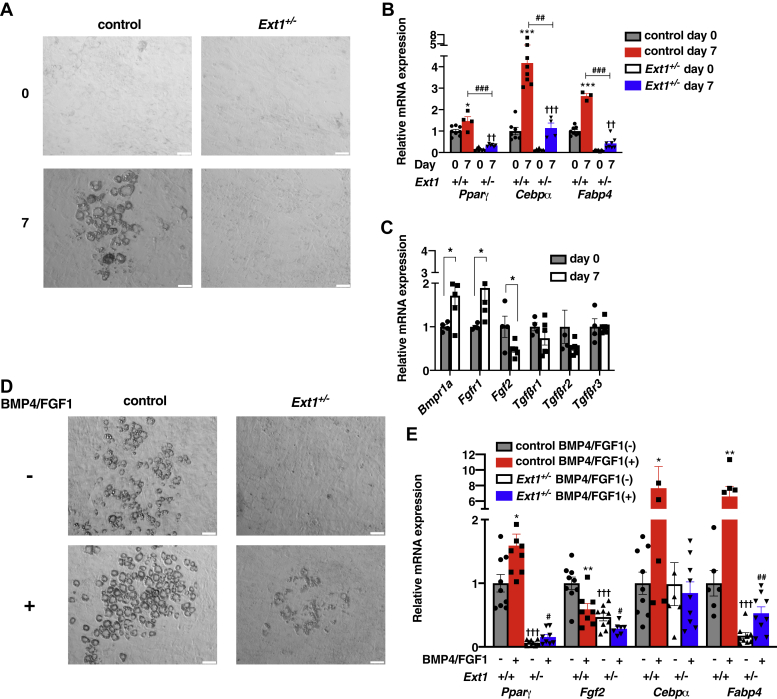
**Heparan sulfate (HS) plays important roles in 3T3-L1 differentiation *via* modulating BMP4–FGF1 signaling.***A*, observation of 3T3-L1 cells at 0 and 7 days after inducing differentiation. The *white* scale bar represents 100 μm. *B*, relative mRNA expression levels of differentiation markers at 0 and 7 days after inducing differentiation. n = 3–8. The mean mRNA expression level of control 3T3-L1 cells at day 0 was set to 1. *Gray*: control cells at day 0; *red*: control cells at day 7; *white*: *Ext1*^+/−^ cells at day 0; *blue*: *Ext1*^+/−^ cells at day 7. ∗Comparison of *gray* and *red*; ^†^comparison of *white* and *blue*; ^#^comparison of *red* and *blue*. *C*, relative mRNA expression levels of differentiation-related genes in control 3T3-L1 cells. n = 4–6. *Gray* and *white bars* indicate the day after inducing differentiation. The mean mRNA expression level at day 0 was set to 1. *D*, observation of 3T3-L1 cells at 7 days after inducing differentiation, with or without BMP4–FGF1 treatment. *Upper pictures*: without BMP4–FGF1 treatment; *lower pictures*: with BMP4–FGF1 treatment. The *white* scale bar represents 100 μm. *E*, relative mRNA expression levels of BMP4–FGF1 signaling and differentiation-related genes. n = 6–9. The mean mRNA expression level of control 3T3-L1 cells without BMP4–FGF1 treatment was set to 1. *Gray*: control cells without BMP4–FGF1 treatment; *red*: control cells with BMP4–FGF1 treatment; *white*: *Ext1*^+/−^ cells without BMP4–FGF1 treatment; *blue*: *Ext1*^+/−^ cells with BMP4–FGF1 treatment. ∗Comparison of *gray* and *red*; ^†^Comparison of *gray* and *white*; ^#^Comparison of *white* and *blue*. ∗^,#^*p* < 0.05; ∗∗^,##^*p* < 0.01; and ∗∗∗^,†††^*p* < 0.005. BMP4, bone morphogenetic protein 4; Bmpr1a, bone morphogenetic protein receptor 1 a; Cebpα, CCAAT/enhancer-binding protein α; Fabp4, fatty acid–binding protein 4; FGF1, fibroblast growth factor 1; Fgfr1, fibroblast growth factor receptor 1; Pparγ, peroxisome proliferator–activated receptor γ; Tgfβr1, TGFβ receptor 1; Tgfβr2, TGFβ receptor 2; Tgfβr3, TGFβ receptor 3.

First, we investigated the expression of enzymes essential for HS biosynthesis during 3T3-L1 differentiation. A prominent increase in *Ext1* expression, in the course of differentiation (Fig. S1*A*), indicates the importance of Ext1-dependent HS synthesis in 3T3-L1 differentiation. To investigate the detailed roles of HS in 3T3-L1 differentiation, we deleted the *Ext1* gene in 3T3-L1 adipocytes using CRISPR–Cas9 technology to obtain *Ext1*-heterodeleted cells (*Ext1*^+/−^ cells) (Fig. S1, *B–D*). Although the differentiation medium induced LD formation in *Ext1*^*+/+*^ 3T3-L1 cells (control cells), no LD formation was observed in *Ext1*^+/−^ cells (Fig. 1*A*). RT-PCR analysis showed reduced expression of the differentiation-related transcription factors peroxisome proliferator–activated receptor gamma (*Pparγ*) and *Cebp*α (Fig. 1*B*). The expression of *acetyl-CoA carboxylase 1*, which encodes a rate-limiting enzyme for adipogenesis (19), was significantly decreased in *Ext1*^+/−^ cells (Fig. S1*E*). Furthermore, the expression of fatty acid–binding protein 4 (*Fabp4*), commonly used as a marker for differentiated adipocytes, was decreased by HS reduction (Fig. 1*B*). To eliminate the possibility that off-target effects of the CRISPR–Cas9 system affected differentiation (20), we evaluated the effect of HSase on the differentiation and impact of *Ext1* overexpression on *Ext1*^+/−^ cells. Microscopic observation and RT-PCR analysis showed the impairment of differentiation and adipogenesis by HSase treatment (Fig. S2). Because transfection efficiency was 70% 1 day after the electroporation and *Ext1* overexpression was transient, we could not completely rescue the phenotype of *Ext11*^+/−^ cells. However, transient *Ext1* overexpression in *Ext1*^+/−^ cells induced LD formation and significantly increased the expression levels of several genes essential for adipogenic differentiation such as *Cebp*α and *Srebp1* (Fig. S3), indicating that impaired phenotypes of *Ext1*^+/−^ were not derived from off-target effects. These results demonstrate that HS reduction in 3T3-L1 cells attenuates their adipogenic differentiation.

### BMP4–FGF1 signaling–mediated adipogenic differentiation was inhibited by HS deletion

Next, we investigated how HS modulates differentiation of preadipocytes into mature adipocytes. Among diverse HSBPs, there is increasing evidence of the importance of bone morphogenetic protein 4 (BMP4) and FGF1 in adipocyte differentiation. BMP4 is essential for the onset of adipocyte differentiation *via* the increased expression of PPARγ, a master regulator for adipocyte differentiation (21, 22). FGF1 inhibits FGF2 expression in adipocytes. Since FGF2 is necessary to maintain preadipocytes in an immature state and to suppress adipogenesis, FGF1-induced FGF2 inhibition facilitates differentiation (23). Thus, we examined the involvement of BMP4–FGF1 signaling pathways in HS-dependent differentiation. Stimulation of 3T3-L1 cells with differentiation medium for 7 days in increased expression of BMP receptor 1a and FGF receptor 1 and reduced expression of Fgf2 (Fig. 1*C*). The expression of receptors for transforming growth factor β (TGF-β), which is an HSBP that modulates adipocyte differentiation (24), was unchanged (Fig. 1*C*), supporting the importance of BMP4–FGF1 signaling pathways in adipocyte differentiation. In normal 3T3-L1 cells, treatment with BMP4–FGF1 facilitated LD enlargement and a robust increase in the expressions of *Pparγ* and *Cebp*α, with decreased expression of *Fgf2* (Fig. 1, *D* and *E*). The impact of BMP4–FGF1 treatment was limited in *Ext1*^+/−^ 3T3-L1 cells, although the treatment promoted differentiation through the upregulation of *Pparγ* and *Fabp4* (Fig. 1, *D* and *E*). These results suggest that BMP4–FGF1 signaling pathways are involved in HS-dependent adipogenic differentiation.

### Glucose uptake by 3T3-L1 cells was attenuated by HS deletion

**Figure 2:**
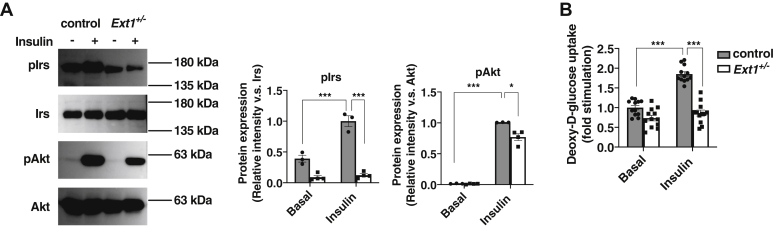
**Insulin-dependent glucose uptake was decreased in *Ext1*-heterozygous knockout 3T3-L1 (*Ext1***^**+/−**^**) cells.***A*, *left*, representative images of Western blot for phosphorylated insulin receptor substrate (pIrs), Irs, pAkt, and Akt, with or without insulin stimulation. *Middle*, protein levels of pIrs corrected by Irs intensity, with or without insulin stimulation. n = 3. *Right*, the protein levels of pAkt corrected by Akt intensity, with or without insulin stimulation. n = 3. The mean intensity of control 3T3-L1 cells with insulin was set to 1. *B*, measurement of deoxy-d-glucose uptake in 3T3-L1 cells, with or without insulin stimulation. *Gray*: control cells, *white*: *Ext1*^+/−^ cells. n = 12. ∗*p* < 0.05 and ∗∗∗*p* < 0.005.

We examined whether the impaired differentiation, caused by HS deletion, would affect glucose uptake by 3T3-L1 cells. Basal expression levels of insulin receptor substrate (Irs) and Akt, which are important proteins for insulin-dependent glucose uptake, did not differ between control and *Ext1*^+/−^ cells (Fig. 2*A*). However, phosphorylation of Irs and Akt by insulin stimulation was significantly impaired in *Ext1*^+/−^ cells (Fig. 2*A*). Moreover, insulin-dependent glucose uptake in *Ext1*^+/−^ cells became half of that measured in control cells (Fig. 2*B*). These *in vitro* experiments demonstrate that HS plays important roles in regulating differentiation and contributes to insulin-dependent glucose uptake.

### HS reduction in mouse WAT resulted in insufficient fat accumulation and lower body weight

Next, we investigated the *in vivo* functions of HS in WATs. A WAT-specific HS-deleted mouse was generated by crossing *Ext1*^*flox/flox*^ mice with *Fabp4-cre* mice, which express Cre recombinase specifically in their WAT. Since homozygous *Ext1* deletion in WAT resulted in embryonic lethality, we produced a male mouse with heterozygous *Ext1* deletion, specifically in WAT (*Fabp4-Cre*^*+*^::*Ext1*^*flox/WT*^ mice, hereafter called *Ext1*^*Δ/WT*^). *Ext1*^*flox/WT*^ mice were used as controls. First, we examined the Cre-dependent DNA recombination of the *Ext1* gene, *Ext1* mRNA expression, and HS levels in visceral (epididymal) WAT (vWAT), because Fabp4 is dominantly expressed in visceral but not subcutaneous WAT (25). *Ext1* gene recombination, decreased *Ext1* mRNA expression, and HS reduction in *Ext1*^*Δ/WT*^ vWAT were confirmed (Fig. S4, *A–D*). No compensatory increase of other HS synthases was observed in vWAT (Fig. S4*B*). We also detected Cre recombinase expression in subcutaneous WAT, brown adipose tissue, and monocytes (26) (Fig. 5*G*). However, *Ext1* mRNA was reduced only in vWAT probably because of low expression levels of Cre recombinase in subcutaneous WAT, brown adipose tissue, and monocytes (Fig. S5, *B*, *D*, and *F*). In addition, previous study showed ectopic *Fabp4* expression of heart, kidney, and liver in mice (27). However, *Ext1* mRNA expressions in these organs were not changed (Fig. S5*H*). These results demonstrated that we obtained mice with HS reduction, specifically in vWAT.

**Figure 3:**
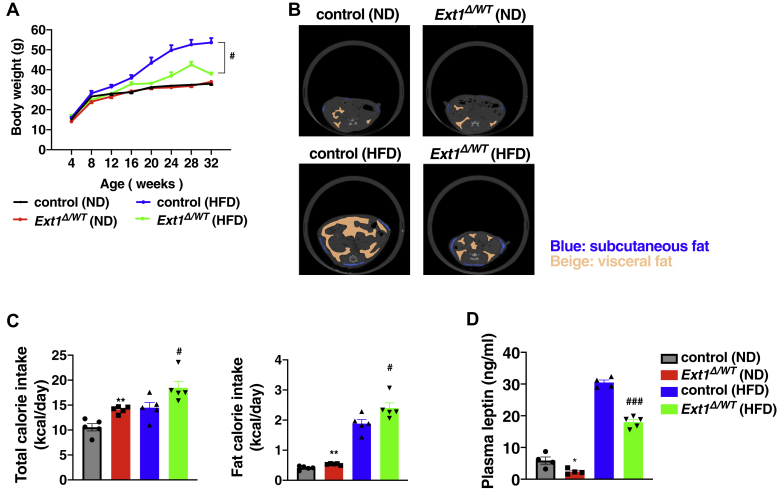
**Body weight and amount of visceral adipose tissue were decreased in *Ext1*-heterozygous deleted mice (*Ext1***^***Δ/WT***^**) fed with HFD.***A*, body weight over time. n = 3–28. *B*, representative images of fat by μCT. *Upper pictures*, control mouse and *Ext1*^*Δ/WT*^ mouse fed with ND; *lower pictures*, control mouse and *Ext1*^*Δ/WT*^ mouse fed with HFD. *Blue*: subcutaneous fat; *beige*: visceral fat. *C*, *left*, total calorie intake per day. *Right*, fat calorie intake per day. n = 5. *D*, plasma leptin concentration 6 h after starvation; *gray*: control mice fed with ND; *red*: *Ext1*^*Δ/WT*^ mice fed with ND; *blue*: control mice fed with HFD; and *green*: *Ext1*^*Δ/WT*^ mice fed with HFD. n = 4–5. ∗Comparison of mice fed ND; ^#^comparison of mice fed HFD. ∗ and ^#^*p* < 0.05, ∗∗*p* < 0.01, and ^###^*p* < 0.005. μCT, micro–computed tomography; HFD, high-fat diet; ND, normal diet.

Body weight and composition did not differ between control and *Ext1*^*Δ/WT*^ mice fed with a normal diet (ND) (Fig. 3, *A* and *B* and Table 1). Although a high-fat diet (HFD) induced robust weight gain with increased adipose tissue in control mice, HFD-fed *Ext1*^*Δ/WT*^ mice exhibited lower body weight with smaller vWAT (Fig. 3, *A* and *B* and Table 1), indicating the involvement of HS in adipogenesis of vWAT, *in vivo*. In addition, higher calorie and fat intakes were observed in *Ext1*^*Δ/WT*^ mice. A lower plasma concentration of leptin, which is released from WAT to suppress appetite, might have contributed to increased food intake (Fig. 3, *C* and *D*).

**Table 1:** Quantification of fat and muscle mass by micro–computed tomography

Genotype	Visceral fat (g)	Subcutaneous fat (g)	Muscle (g)	Body weight (g)
Control (ND)	0.11 ± 0.01	2.04 ± 0.03	15.28 ± 0.56	24.49 ± 0.45
*Ext1*^*Δ/WT*^ (ND)	0.08 ± 0.01	1.93 ± 0.01	14.21 ± 0.38	24.95 ± 0.34
Control (HFD)	1.12 ± 0.01	2.57 ± 0.15	20.77 ± 0.33	31.65 ± 0.49
*Ext1*^*Δ/WT*^ (HFD)	0.45 ± 0.10^a^	2.24 ± 0.18	20.20 ± 0.39	29.65 ± 0.45^b^

Eight-week-old mice were used in this experiment. Comparison of mice fed HFD. n = 4–6.

a *p* < 0.005.

b *p* < 0.01.

### Impaired differentiation and adipogenesis in *Ext1*^*Δ/WT*^ adipocytes

**Figure 4:**
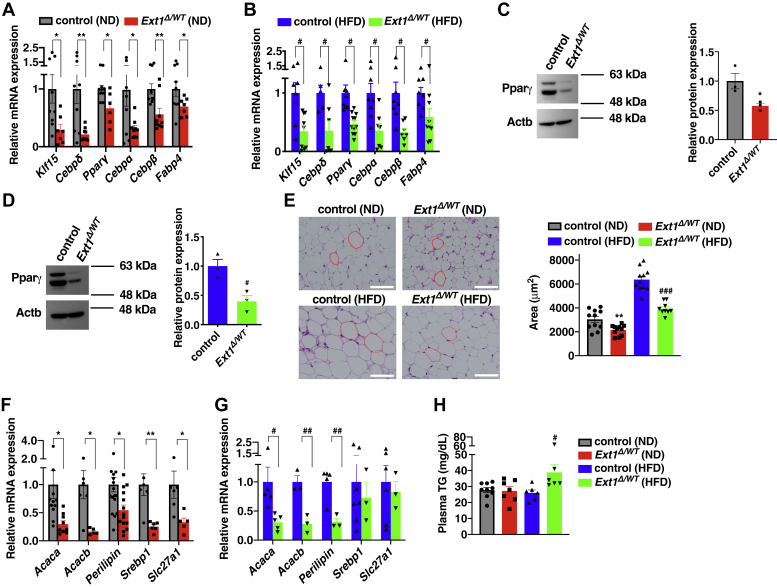
**The differentiation of visceral white adipocytes was attenuated in *Ext1***^***Δ/WT***^**mice of epididymal white adipose tissue.***A* and *B*, relative mRNA expression levels of differentiation markers in vWAT. n = 5–11. *C* and *D*, *left*, representative images of Western blot in Pparγ. *Right*, relative protein level of Pparγ in vWAT. n = 3. The mean Pparγ expression level in control vWAT was set to 1. *E*, *left*, observation of vWAT by H&E staining. The *white* scale bar represents 50 μm. *Right*, the averaged area of adipocytes in upper pictures. n = 9–11. At least 100 areas were measured, per mouse. *F* and *G*, relative mRNA expression levels of adipogenic genes in vWAT. n = 3–18. In *A*, *B*, *F*, and *G*, the mean mRNA expression level of control vWAT was set to 1. *H*, plasma triglyceride concentration 6 h after starvation. n = 6–9. *Gray*: control mice fed with ND; *red*: *Ext1*^*Δ/WT*^ mice fed with ND; *blue*: control mice fed with HFD; and *green*: *Ext1*^*Δ/WT*^ mice fed with HFD. ∗Comparison of mice fed ND; ^#^Comparison of mice fed HFD. ∗ and ^#^*p* < 0.05; ∗∗ and ^##^*p* < 0.01. Acaca, acetyl-CoA carboxylase alpha; Acacb, acetyl-CoA carboxylase beta; *Cebpδ*, CCAAT/enhancer-binding protein δ; *Cebpβ*, CCAAT/enhancer-binding protein β; HFD, high-fat diet; *Klf15*, krüppel-like factor 15; ND, normal diet; Pparγ, peroxisome proliferator–activated receptor gamma; Slc27a1, long-chain fatty acid transport protein 1; Srebp1, sterol regulatory element–binding protein 1; vWAT, visceral white adipose tissue.

We investigated the impact of HS reduction on vWAT differentiation. The expression levels of transcriptional factors, including *Klf15* and *Cebp*α, and the differentiation marker *Fabp4* were decreased in *Ext1*^*Δ/WT*^ vWAT (Fig. 4, *A* and *B*). We also investigated the level of protein expression of the master differentiation regulator, Pparγ, and confirmed that this was reduced in *Ext1*^*Δ/WT*^ vWAT (Fig. 4, *C* and *D*). H&E staining showed that the visceral white adipocytes of *Ext1*^*Δ/WT*^ mice were smaller than that of control mice, regardless of diet (Fig. 4*E*). Several factors of adipogenesis, such as *acetyl-CoA carboxylase 1*, *acetyl-CoA carboxylase 2*, and *perilipin* (19), were reduced in *Ext1*^*Δ/WT*^ vWAT (Fig. 4, *F* and *G*). These results indicate that HS reduction impairs adipocyte differentiation and results in the formation of small adipocytes with lower adipogenesis and lipid storage. The lower capacity of lipid storage in visceral white adipocytes of *Ext1*^*Δ/WT*^ mice might contribute to the higher plasma triglyceride levels found in *Ext1*^*Δ/WT*^ mice fed with HFD (Fig. 4*H*). In addition, we did not observe the alteration of subcutaneous white adipocytes and brown adipocytes between two groups (Fig. S6).

**Figure 5:**
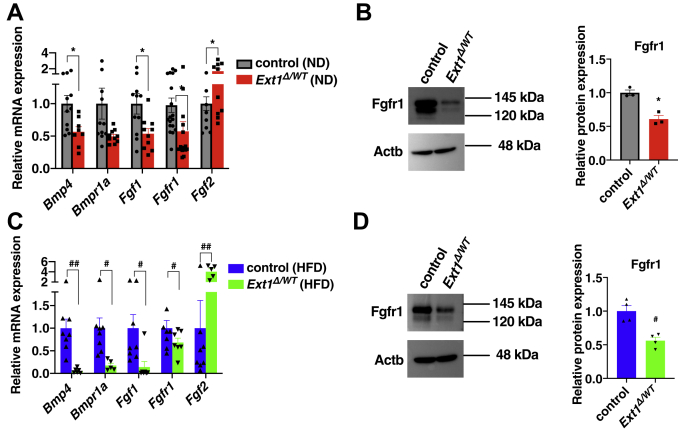
**Expressions of BMP4–FGF1 signaling molecules were altered in vWAT of *Ext1***^***Δ/WT***^**mice.***A* and *C*, relative mRNA expression levels of BMP4–FGF1 pathway–related genes in vWAT. n = 5–18. The mean mRNA expression level of control vWAT was set to 1. *B* and *D*, *left*, representative images of Western blot in Fgfr1. *Right*, relative protein level of Fgfr1 in vWAT. n = 3. The mean Fgfr1 expression level of control WAT was set to 1. We intentionally showed the same Western blot images of Actb for Figures 4*C* and 5*B* because we used same adipose tissues for Western blotting in these experiments. We also showed the same Actb images for Figures 4*D* and 5*D* because we used same adipose tissue homogenates to examine the expression levels of Pparγ (Fig. 4*D*) and Fgfr1 (Fig. 5*D*). *Gray*: vWAT isolated from control mice fed with ND; *red*: vWAT isolated from *Ext1*^*Δ/WT*^ mice fed with NDl; *blue*: vWAT isolated from control mice fed with HFD; and *green*: vWAT isolated from *Ext1*^*Δ/WT*^ mice fed with HFD. ∗Comparison of mice fed with ND; ^#^Comparison of mice fed with HFD. ∗ and ^#^*p* < 0.05; ^##^*p* < 0.01. BMP4, bone morphogenetic protein 4; FGF1, fibroblast growth factor 1; Fgfr1, fibroblast growth factor receptor 1; HFD, high-fat diet; ND, normal diet; Pparγ, peroxisome proliferator–activated receptor gamma; vWAT, visceral white adipose tissue.

We further examined the effect of HS deletion on BMP4–FGF1 signaling pathways in vWAT. RT-PCR analysis showed decreased mRNA expression of the ligands and their receptors and a significant increase in *Fgf2* expression in *Ext1*^*Δ/WT*^ vWAT (Fig. 5), indicating that HS inhibition affected BMP4–FGF1 signaling pathways and led to attenuated adipogenic differentiation.

### Glucose intolerance because of insulin resistance in *Ext1*^*Δ/WT*^ mice

**Figure 6:**
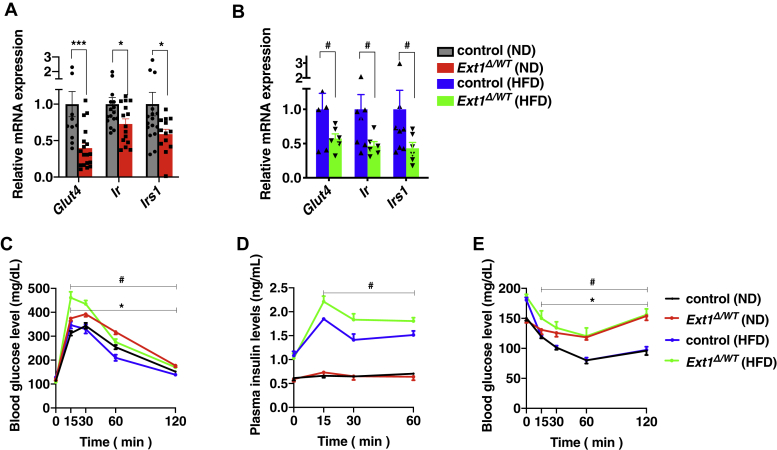
***Ext1***^***Δ/WT***^**mice showed impaired glucose tolerance because of insulin resistance.***A* and *B*, relative mRNA expression levels of glucose uptake–related genes in vWAT. n = 6–18. The mean expression level of control vWAT was set to 1. *C*, blood glucose levels in mice after intraperitoneal glucose injection. n = 8–27. *D*, plasma insulin concentrations after intraperitoneal injection of glucose. n = 4–9. *E*, blood glucose levels in mice after intraperitoneal insulin injection: n = 8–13. *Gray*: control mice fed with ND; *red*: *Ext1*^*Δ/WT*^ mice fed with ND; *blue*: control mice fed with HFD; and *green*: *Ext1*^*Δ/WT*^ mice fed with HFD. ∗Comparison of mice fed with ND; ^#^Comparison of mice fed with HFD. ∗ and ^#^*p* < 0.05; ∗∗∗*p* < 0.005. Glut4, glucose transporter 4; HFD, high-fat diet; Ir, insulin receptor; Irs1, insulin receptor substrate 1; ND, normal diet; vWAT, visceral white adipose tissue.

We examined the involvement of HS in glucose homeostasis. The mRNA expression levels of *glucose transporter 4*, *insulin receptor*, and *Irs1*, which encode essential proteins for insulin-dependent glucose uptake, were reduced in *Ext1*^*Δ/WT*^ vWAT (Fig. 6, *A* and *B*). Upon glucose challenge, *Ext1*^*Δ/WT*^ mice fed with ND showed significantly higher glucose levels (Fig. 6*C*). When stressed by HFD, aggravated glucose intolerance was observed in *Ext1*^*Δ/WT*^ mice. Although plasma insulin levels were not decreased in *Ext1*^*Δ/WT*^ mice compared with control mice (Fig. 6*D*), the hypoglycemic action of insulin was impaired in *Ext1*^*Δ/WT*^ mice, according to the insulin tolerance test (ITT; Fig. 6*E*). These results revealed that *Ext1*^*Δ/WT*^ mice had glucose intolerance because of insulin resistance, with lower expression levels of insulin-signaling molecules in white adipocytes.

## Discussion

In this study, we investigated the role of HS in 3T3-L1 adipocytes and mouse vWAT. Our experiments using 3T3-L1 cells demonstrate that HS is required for their differentiation *via* the induction of adipogenic transcriptional factors and contributes to normal insulin-dependent glucose uptake. *In vivo* experiments reveal that HS in vWAT induces differentiation and plays an important role in normal insulin sensitivity. It is noteworthy that *Ext1*^*Δ/WT*^ mice fed with ND develop glucose intolerance because of insulin resistance in the absence of the detrimental effects of HFD on metabolic organs, emphasizing the critical importance of HS in vWAT for glucose homeostasis. A possible contribution of BMP4–FGF1 signaling pathways to HS-dependent adipocyte differentiation is also indicated.

We analyzed a male mouse with heterozygous *Ext1* deletion, specifically in WAT because of lethality of homozygous *Ext1* deletion in WAT. Systemic *Ext1* homozygous deficiency led to embryonic lethality because of failure of gastrulation, lack of organized mesoderm, and extraembryonic tissues at embryonic day 8.5 (E8.5) (4), demonstrating that HS is essential for embryonic development. In the present study, we could confirm that *Fabp4-Cre*^*+*^::*Ext1*^*flox/flox*^ mice were alive at E16.5 (Fig. S7). Previous study has shown that *Fabp4* is also expressed in the heart, kidney, and liver (27), and Cre expression is detected in cardiomyocytes and hepatocytes of *Fabp4-Cre*^*+*^ mice (28). Indeed, we confirmed that the Cre expression in the heart, kidney, and liver of *Fabp4-Cre*^*+*^ mice were detected at E16.5 (Fig. S7). In addition, Wang *et al.* (29) reported that vWAT developed postnatally. These results might indicate that *Fabp4-Cre*^*+*^::*Ext1*^*flox/flox*^ mice might die of cardiac, hepatic, and/or renal dysfunction from E16.5 to P0, although further research is required to determine the responsible organ for embryonic lethality of *Fabp4-Cre*^*+*^::*Ext1*^*flox/flox*^ mouse.

*Ext1* mRNA was decreased in vWAT but not in subcutaneous WAT. Although both WATs have a large capacity to store excessive energy in the form of triglycerides, they have different developmental origins and distinct characteristics (30). On the one hand, transplantation of subcutaneous WAT can ameliorate metabolic dysregulation (31). On the other hand, an excess of vWAT is associated with insulin resistance, and removal of vWAT during bariatric surgery improves insulin sensitivity (32). Our study also showed the strong contribution of vWAT to systemic glucose homeostasis. These evidences indicate the contradictory impact of these WATs on energy homeostasis. Since the importance of HS in subcutaneous WAT was not evaluated in this study, further studies should examine its role in differentiation and energy homeostasis.

*Ext1*^*Δ/WT*^ mice showed glucose intolerance because of insulin resistance and demonstrates the strong contribution of vWAT to systemic glucose homeostasis. One of the limitations of our study was that we could not evaluate the importance of HS in subcutaneous WAT. Further studies are required to examine its role in differentiation and energy homeostasis.

Glucose intolerance because of insulin resistance, the metabolic phenotype induced by HS deletion in vWAT, is usually found in obese subjects with metabolic syndrome. However, *Ext1*^*Δ/WT*^ mice fed with HFD had a lean phenotype, with decreased visceral fat mass and increased food intake. These characteristics are also observed in other diabetic animal models. Attenuated adipocyte differentiation because of the disruption of transcription factors can result in decreased energy storage, smaller fat mass, and glucose intolerance (17, 33). Lipoatrophic diabetes, caused by abnormal lipid homeostasis, results in less lipid storage in the adipose tissues and voracious appetite, with lower leptin secretion, higher blood triglyceride levels, and insulin resistance (33, 34). In the present study, we confirmed no ectopic accumulation of fat in liver and muscles (Fig. S8, *A–C*). In addition, we examined home cage locomotor activity test to assess whether increased activity of *Ext1*^*Δ/WT*^ mice resulted in elevated energy expenditure. However, locomotor activity was not changed between two groups (Fig. S8*D*), indicating that energy expenditure might not be altered. Therefore, it can be deduced that HS deletion impairs adipocyte differentiation, leading to insufficient capacity for energy storage and a spillover of excessive glucose and triglyceride into the blood, without inducing obesity.

The importance of BMP4 and FGF1 in adipocyte differentiation and insulin sensitivity has been clearly demonstrated by various studies (21, 22, 23, 35). In our *in vitro* study, BMP4–FGF1-induced adipogenic differentiation was inhibited by HS deletion in 3T3-L1 cells. Moreover, our *in vivo* study revealed that a significant reduction in Bmp4, Fgf1, and their receptors was induced by HS deletion in *Ext1*^*Δ/WT*^ mice. These data indicate substantial interaction of HS with BMP4–FGF1 signaling pathways in the differentiation of adipocytes and insulin sensitivity. However, we could not rule out the possibility that other HS-dependent signaling pathways also affected metabolic phenotypes. Treatment of *Ext1*^+/−^ 3T3L1 cells with exogenous BMP4–FGF1 had a limited effect on adipogenic differentiation. Previous studies have shown that BMP4 or FGF1 deficiency in mice induces various phenotypes, such as adipocyte enlargement or macrophage infiltration, although these studies were concerned with the impact of whole-body homozygous deletion of the ligands (35, 36). Several reports have shown the inhibitory effect of TGF-β, one of the HSBPs, on adipocyte differentiation (24). Although we examined the involvement of TGF-β pathways by evaluating the expression levels of three TGF-β receptors, HS deletion in 3T3-L1 cells, and vWAT did not alter their expression (Fig. S9, *A–C*). Previous reports have shown that an HS proteoglycan betaglycan (TGF-β receptor III) is involved in TGF-β–dependent adipogenic differentiation (37). TGF-β could bind to the core protein betaglycan as well as to HS chains. Indeed, TGF-β stimulation induced phosphorylation of Smad3 in 3T3-L1 cells regardless of HS amount. (Fig. S9*D*). Thus, the interaction between TGF-β and core protein betaglycan, but not HS chains, might be sufficient to transduce their signals; however, further studies are required to examine the involvement of HS in TGF-β signaling pathways in adipocytes. Wnt ligands also have a significant impact on the differentiation of various tissues (38). In WAT, the canonical pathway inhibits adipogenesis, and the noncanonical pathway induces it (39, 40). HS 6-O-sulfation, which is accelerated by HS-6-sulfotransferase and removed by glucosamine-6-sulfatases (Sulf1 and Sulf2), determines the preference for canonical or noncanonical pathways. In *Xenopus*, Sulf1 overexpression inhibits the canonical pathway and enhances the noncanonical pathway (41). In muscle cells, deletion of Sulf1 and Sulf2 promotes the noncanonical pathway (42). Since Ext1 deletion results in the attenuation of both signaling pathways, further research focusing on HS-6-sulfotransferase, Sulf1, and Sulf2 will clarify the importance of fine tuning the HS chains in adipogenic differentiation. The identified binding ligands of HS include growth factors, extracellular matrix components, cell–cell adhesion molecules, lipoproteins, cytokines, chemokines, and coagulation factors (2). Therefore, it is conceivable that several HS-dependent signaling pathways cooperatively regulate adipocyte differentiation. Further studies are required to investigate the detailed molecular mechanisms of HS-dependent adipocyte differentiation.

The PPARγ agonists pioglitazone and rosiglitazone are widely used to ameliorate insulin resistance in patients with type II diabetes. Although these agonists affect energy homeostasis *via* pleiotropic action on various tissues, several reports have revealed that their induction of adipocyte differentiation plays a role in lowering blood glucose (43, 44). This raises the idea that promotion of adipocyte differentiation would be an effective therapy against insulin resistance. Recent preclinical studies have shown that *BMP4* gene therapy, using adeno-associated virus 8, enhances insulin sensitivity (45), and that exogenous FGF1 administration sensitizes the tissue to insulin (46). Our study has shown the significant contribution of HS to adipocyte differentiation through BMP4–FGF1 signaling pathways and insulin sensitivity. In addition, our previous study revealed the involvement of HS in pancreatic β-cell differentiation and insulin secretion (14). Taken together, HS could be a promising target for drug development to improve insulin resistance *via* BMP4–FGF1-dependent adipogenic differentiation, and also to increase insulin secretion from β-cells. Therefore, it may be worthwhile to examine the therapeutic potential of increasing HS on metabolic organs and develop novel drugs that increase HS itself and/or HS-dependent signaling pathways.

In conclusion, we generated *Ext1*-heterozygous knockout 3T3-L1 cells (*Ext1*^+/−^ cells) and visceral white adipocyte–specific heterozygous *Ext1*-deleted mice (*Ext1*^*Δ/WT*^ mice) to investigate the importance of HS in white adipocytes. Our results demonstrate that HS plays a crucial role in the differentiation of white adipocytes, thereby contributing to normal insulin sensitivity and glucose homeostasis. Overall, these findings could improve our understanding of diabetes and lead to the development of novel therapies for diabetes by targeting HS.

## Experimental procedures

### Cell culture and adipocyte differentiation

The 3T3-L1 cells (American Type Culture Collection) were maintained in Dulbecco's modified Eagle's medium (DMEM; Wako Pure Chemical Industries, Ltd) supplemented with 10% bovine calf serum (GE Healthcare) and 5% CO_2_ at 37 °C. At 2 days after confluence (day 0), differentiation was induced by exchanging the previous medium for DMEM containing 10% fetal bovine serum (GE Healthcare), 1 μg/ml insulin (Wako), 0.25 μM dexamethasone (Nacalai Tesque, Inc), and 0.5 mM 3-isobutyl-1-methylxanthine (Nacalai), for 48 h. At day 2, the culture medium was changed with DMEM containing 10% fetal bovine serum and 1 μg/ml insulin. At days 4 and 6, the culture medium was changed with DMEM containing 10% fetal bovine serum (47). At day 7, we observed LDs using an inverted microscope (IX-71; Olympus Life Science).

### Heterozygous deletion of the *Ext1* gene from 3T3-L1 cells (*Ext1*^+/−^ cells)

The CRISPR–Cas9 system was introduced to generate *Ext1*-heterozygous deleted 3T3-L1 (*Ext1*^+/−^) cells. The oligonucleotides corresponding to *Ext1* exon 1, containing the protospacer adjacent motif sequence, were inserted into the BbsI restriction site of the pX459 v2.0 vector (plasmid number #62988; Addgene). The guide RNA sequences used in this study were designated using CRISPR direct (48) and are shown in Table S1. Transfection was performed using Lipofectamine 2000 (Thermo Fisher Scientific) and Opti-MEM (Thermo Fisher Scientific) according to the manufacturer's protocol. Briefly, 2.5 × 10^5^ cells per well were seeded in a 6-well plate before transfection and incubated overnight with 250 μl of a mixture of Lipofectamine 2000 and Opti-MEM. After that, we selected the puromycin-resistant stable transformants for further culture. After the dideoxy sequencing of isolated genomic DNA from the transformants to confirm the genome editing, *Ext1*^+/−^ cells were established.

### Quantitative RT-PCR

RNA isolation, RT, and real-time PCR were performed, as described previously (49). Gene expression levels were normalized against beta-actin expression. The primers used in this study are shown in Table S2.

### Immunocytochemistry

3T3-L1 cells seeded in a 6-well plate were washed with PBS and fixed with methanol (Wako)/ethanol (Wako) at −20 °C for 15 min. After blocking with PBS containing 0.5% bovine serum albumin (Nacalai) and 0.05% sodium azide (Wako), 3T3-L1 cells were incubated with primary antibody at 4 °C overnight. After washing, cells were incubated with fluorescent dye–conjugated secondary antibody for 1 h at room temperature. The antibodies used in this study are shown in Table S3. Nuclei were stained with Hoechst 33258 (Dojindo). Images were captured using a confocal microscope C2si (Nikon).

### Heparinase treatment of 3T3-L1 cells

To pharmacologically remove HS from 3T3-L1 cells, we treated 3T3-L1 cells by heparinase III (HSase, 40 mU/ml; New England Biolabs), which degrades HS (50), as described previously with slight modification (51). HSase treatment was performed at day 0, day 2, day 4, and day 6.

### BMP4 and FGF1 stimulation

3T3-L1 cells were incubated with BMP4 (40 ng/ml; Wako) for 2 days (from day −2 to day 0). After that, we induced differentiation as described previously. FGF1 (10 ng/ml; Miltenyi Biotec) was added to the differentiation medium.

### Transient *Ext1* overexpression

3T3-L1 cells were electroporated as follows. First, we mixed 1.0 × 10^6^ 3T3-L1 cells and 20 μg of pCI-neo mammalian expression vector (Promega) with or without mouse *Ext1* complementary DNA in 200 μl of Opti-MEM (Thermo Fisher Scientific). After these complexes were transferred to an electroporation cuvette, electroporation was performed using CUY21EDIT II (Bex Co, Ltd) according to the manufacturer's protocol. After that, we seeded the cells at 24-well plate and induced differentiation as described previously.

### Insulin and TGF-β stimulation

At 7 days after differentiation, 3T3-L1 cells were washed with Tris-buffered saline and stimulated in a culture medium containing insulin (50 nM) and TGF-β (1 ng/ml; Peprotech), respectively, for 30 min. After that, proteins were extracted using radioimmunoprecipitation assay buffer (50 mM Tris–HCl buffer, pH 7.6, 150 mM NaCl, 1% NP-40, 0.5% sodium deoxycholate, and 0.1% SDS) containing sodium orthovanadate (Wako).

### Western blotting

Isolated adipose tissues were lysed with radioimmunoprecipitation assay buffer. The levels of proteins were measured by bicinchoninic acid assay, as described previously (52). Aliquots containing 10 μg protein were separated on 8% or 12% SDS-polyacrylamide gels, and the proteins were then transferred to polyvinylidene difluoride membranes, which were blocked with 5% nonfat dry milk solution for 1 h. The membranes were incubated with primary antibodies at 4 °C overnight and then incubated with secondary antibodies at room temperature for 1 h. The antibodies used in this study are shown in Table S3. Immunoreactive bands were detected using an EzWestLumi plus (ATTO), and bands were recorded using the Ez-capture MG chemiluminescence imaging system (ATTO). The intensity levels were calculated using ImageJ (National Institutes of Health [NIH]) (53).

### Glucose uptake by the 3T3-L1 adipocytes

We used 3T3-L1 cells at 7 days after differentiation and incubated them in serum-free DMEM medium 2 h before the assay. The assay was performed as described previously (54, 55). Briefly, the cells were washed several times with no-glucose DMEM (Nacalai) and treated with 1 μM insulin for 10 min at 37 °C. Then, the cells were incubated at 37 °C in no-glucose DMEM with 0.5 μCi of [^3^H]-2-deoxy-d-glucose (PerkinElmer) and 5 mM of unlabeled 2-deoxy-d-glucose (Nacalai) for 10 min. After that, the cells were washed with ice-cold perfusion in PBS containing 1 mM CaCl_2_ and MgCl_2_ and lysed with 100 mM NaOH. Finally, the lysates were mixed with 2 ml of liquid scintillator cocktail (Ultima Gold XR; PerkinElmer), and the radioactivity was measured with an LSC-1000 (Hitachi Aloka Medical). Protein concentrations were measured using a Coomassie Protein Assay Kit (Thermo Fisher Scientific).

### Mice

All mice used in this study were handled in accordance with the Principles for the use of Research Animals of Tohoku University (2019MdA-327-03 and 2019MdLMO-203-02). All mice were kept under a 12-h light/dark cycle (8:00 AM/8:00 PM) in a humidity- and temperature-controlled room, and water and food were provided ad libitum. Labo MR Stock (3.29 kcal/g, crude fat: 3.9%; NOSAN Yokohama) and Quick FAT (3.96 kcal/g, crude fat: 13%; CLEA Japan, Inc) were used as ND and HFD, respectively. The creation of the loxP-modified *Ext1* allele (Ext1 flox) (6) and *Fabp4-Cre* transgenic mice (The Jackson Laboratory) has been previously described (56). In this experiment, 8- to 12-week-old male mice were used, unless otherwise noted.

### Immunohistochemistry

The WAT was quickly removed after perfusion of PBS and 4% paraformaldehyde (Wako). After isolation of WAT, WAT was further fixed in 10% formalin neutral buffer solution (Wako). After embedding in paraffin and making sections of 8 μm thicknesses, sections were blocked with 10% normal goat serum (Nichirei Biosciences, Inc) for 15 min at room temperature. Then, sections were incubated with primary antibodies overnight at 4 °C. After washing with PBS, sections were incubated with secondary antibody for 2 h at room temperature. The antibodies used in this study are shown in Table S3. Finally, sections were mounted with Vectashield Hardset with 4′,6-diamidino-2-phenylindole (Vector Laboratories). Images were captured using a confocal microscope C2si (Nikon).

### Monocytes separation

Blood samples were collected from abdominal aorta with heparin sodium treatment (Mochida Pharmaceutical Co, Ltd). After that, monocytes were separated using lymphocyte separation solution (Nacalai) with modifications (57).

### Micro-computed tomography analysis of adipose tissue

Body fat composition was analyzed as described previously, with some modifications (58). In brief, mice were sacrificed and scanned between the proximal end of the first vertebra and the distal end of the tibia, using the Latheta LCT-200 experimental animal X-ray CT system (micro–computed tomography; Hitachi). Tail, feet, and head were excluded because they contain negligible amounts of fat. The voxel size was 96 × 96 μm, and Latheta software was used to characterize the volume and mass of adipose tissues.

### Glucose tolerance test and ITT

Glucose (Wako; 2 g/kg body weight) and insulin (0.75 U/kg body weight) were injected intraperitoneally. Both tests were performed as described previously (14). Blood samples were taken from the tail. The blood glucose concentrations were determined with an ACCUCHEK comfort blood glucose analyzer (Roche Ltd).

### Measurement of the concentration of HS in WAT

Isolated adipose tissues were incubated with 0.5 M Tris–HCl (pH 7.5) containing actinase E (Kaken Pharmaceutical Co, Ltd; 2 mg/ml) at 55 °C overnight. After that, the levels of HS were determined using the HS ELISA kit (Seikagaku Biobusiness Corporation).

### Measurement of plasma insulin and leptin

The concentrations of insulin and leptin were determined by ELISA (insulin; Morinaga, leptin; Shibayagi). Blood samples were taken from the tail vein. For leptin measurement, mice were fasted for 6 h before the experiment.

### Measurement of adipocyte size by H&E staining

After perfusion and fixation, H&E staining was performed as described previously (59). A BZ-9000 Fluorescence Microscope (Keyence) was used for taking the images, and ImageJ software (NIH) was used for morphometric analysis. The areas of white adipocytes were calculated using ImageJ (53).

### Oil red O staining

The liver, tibialis anterior muscle, and gastrocnemius muscle were quickly removed after perfusion with PBS and then was fixed in 4% paraformaldehyde (Wako). After isolation of these tissues, they were fixed in 10% formalin neutral buffer solution (Wako). Oil red O staining was performed as described previously with some modifications (60). A BZ-9000 Fluorescence Microscope was used for observation, and the Hybrid Cell Count image analysis program (Keyence) was used for morphometric analysis.

### Home cage locomotor activity

Home cage locomotor activity was assessed as described previously (61). Mice were transferred to individual home cage and habituated for 24 h prior to the recording of locomotor activity. Locomotor activity was measured using an activity-monitoring system with infrared-beam apparatus (SUPERMEX; Muromachi Kikai Co Ltd). Data were collected and analyzed using CompACT AMS software (Muromachi Kikai Co Ltd).

### Statistical analysis

Experiments were analyzed using GraphPad Prism 8 (GraphPad Software) for statistical analysis. Student's *t* tests were utilized, except for body weights, glucose tolerance test, plasma insulin levels, and ITT, which were analyzed using two-way ANOVA. A *p* value <0.05 was regarded as significant. Data are presented as means ± SEM.

## Data availability

The data used to support the findings of this study are available from the corresponding author upon request.

## Supporting information

This article contains supporting information.

## Conflict of interest

The authors declare that they have no conflicts of interest with the contents of this article.
