# Supplementary material for: Heparan sulfate promotes differentiation of white adipocytes to maintain insulin sensitivity and glucose homeostasis
Source: J Biol Chem. 2021 Jul 24;297(3):101006. doi: 10.1016/j.jbc.2021.101006 (PMC8379462; doi:10.1016/j.jbc.2021.101006)
Supplement: Figures S1–S9 and Tables S1–S3 [file mmc1.pdf]

Supplementary Materials

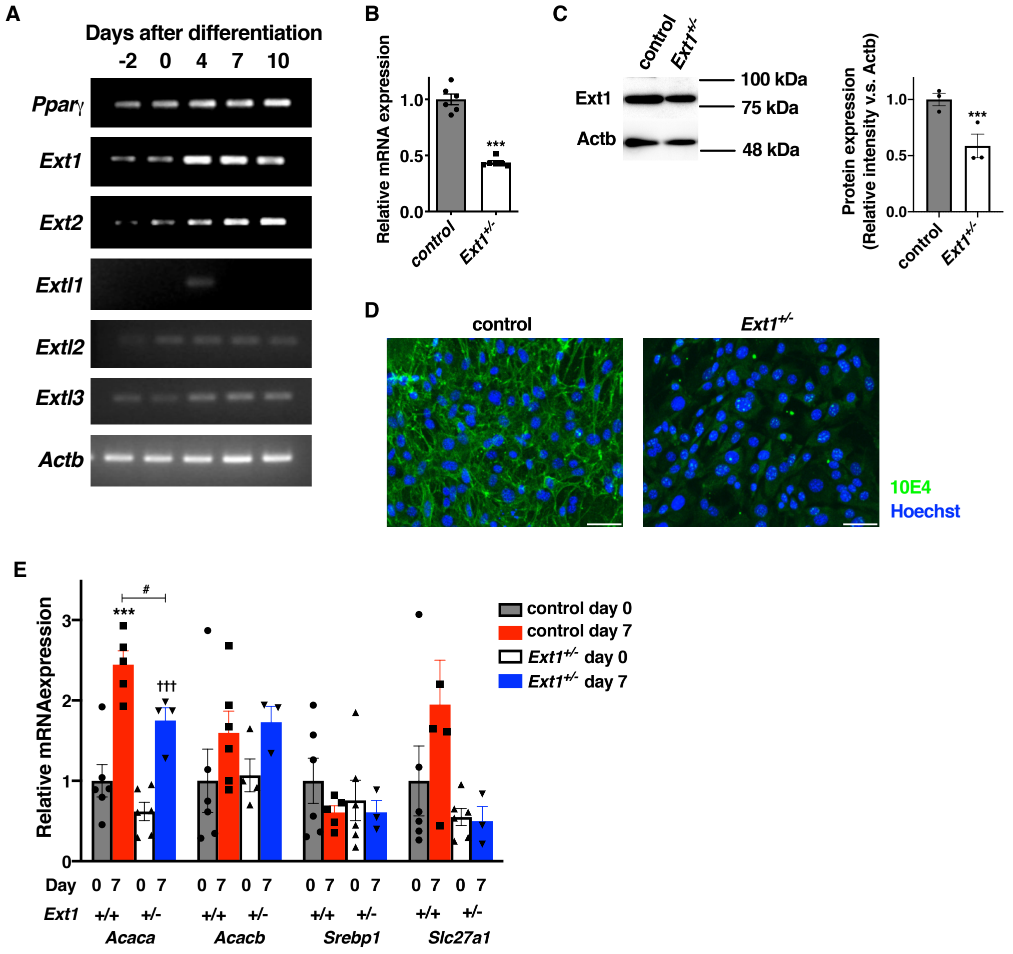

Suppl. Fig. 1. Confirmation of *Ext1*-hetero deletion in 3T3-L1 cells (*Ext1*$^{+/-}$ cells).

(A) mRNA expression levels of heparan sulfate (HS) synthases in 3T3-L1 cells during differentiation. (B) and (C) Generation of the *Ext1*-heterozygous knockout 3T3-L1 cells (*Ext1*$^{+/-}$ cells) by CRISPR-Cas9 system. (B) Relative mRNA expression levels of *Ext1*. n = 6. The mean mRNA expression level of control 3T3-L1 cells was set to 1. (C) Left: representative images of western blot in Ext1. Right: relative protein levels of Ext1. The mean Ext1 expression level in control 3T3-L1 cells was set to 1. n = 3. (D) Immunocytochemical staining for HS (10E4: green) and Hoechst (blue) in 3T3-L1 cells. White scale bar: 50 µm. (E) Relative mRNA expression levels of adipogenesis-related genes at days after inducing differentiation. n = 3 ~ 6. Gray: control cells at day 0, red: control cells at day 7, white: *Ext1*$^{+/-}$ cells at day 0, blue: *Ext1*$^{+/-}$ cells at day 7. The mean mRNA expression level of control 3T3-L1 cells at day 0 was set to 1. * comparison of gray and red, † comparison of white and blue, # comparison of red and blue. $^{\#}p < 0.05$, $^{***,\dagger\dagger\dagger}p < 0.005$.

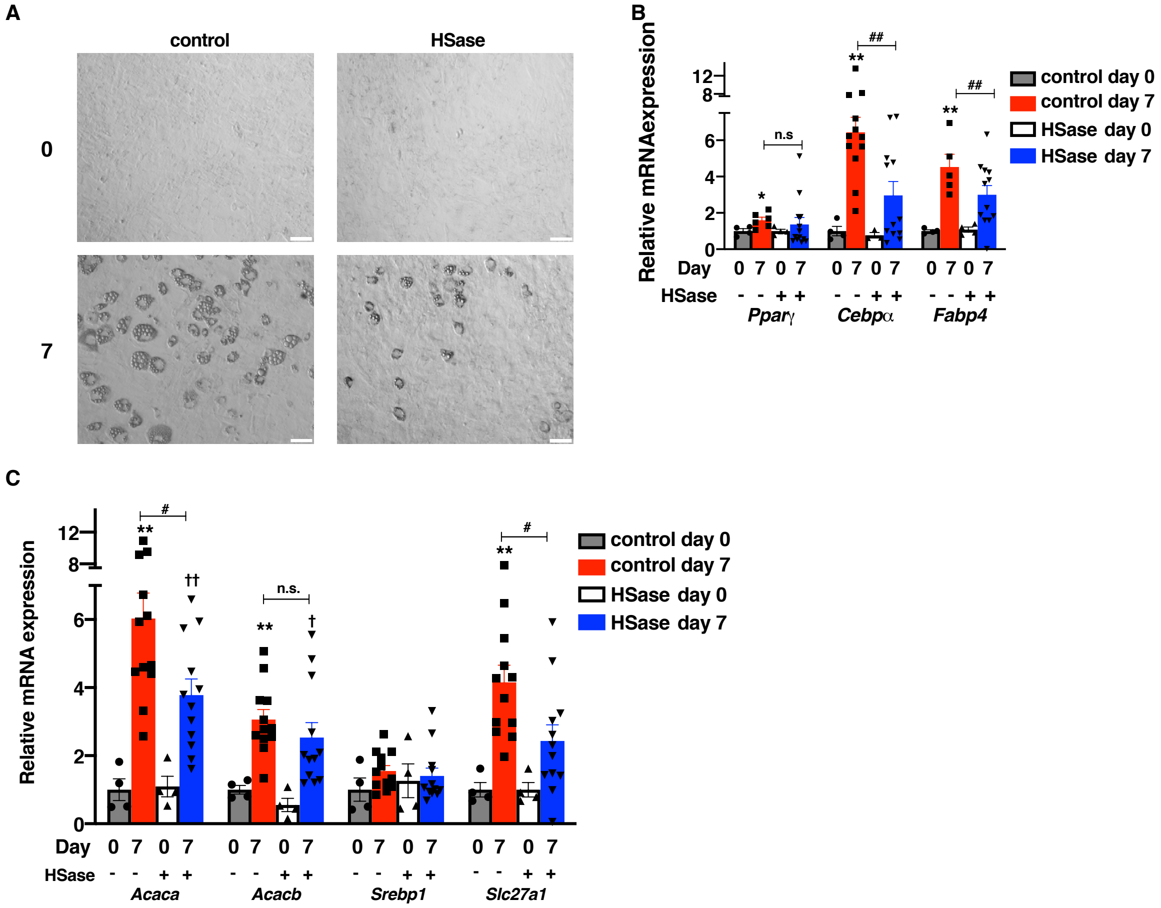

Suppl. Fig. 2. Inhibitory effect of enzymatic removal of HS on 3T3-L1 cells differentiation.

(A) Observation of 3T3-L1 cells at 0 and 7 days after inducing differentiation. control: 3T3-L1 cells without heparinase treatment. HSase: 3T3-L1 cells with heparinase treatment. White scale bar: 100 μm. (B) Relative mRNA expression levels of differentiation markers at 0 and 7 days after inducing differentiation. n = 3 ~ 13. (C) Relative mRNA expression levels of adipogenesis-related genes. n = 4 ~ 12. Gray: 3T3-L1 cells without heparinase treatment at 0 day, red: 3T3-L1 cells without heparinase treatment at 7 days, white: 3T3-L1 cells with heparinase treatment at 0 day, blue: 3T3-L1 cells with heparinase treatment at 7 days. The mean mRNA expression level of 3T3-L1 cells without heparinase treatment at 0 day was set to 1. * comparison of gray and red, † comparison of whie and blue, $^{\#}$ comparison of red and blue. $^{*,\#,\dagger}$ $p < 0.05$, $^{**,\#\#,\dagger\dagger}$ $p < 0.01$, n.s.: not significant.

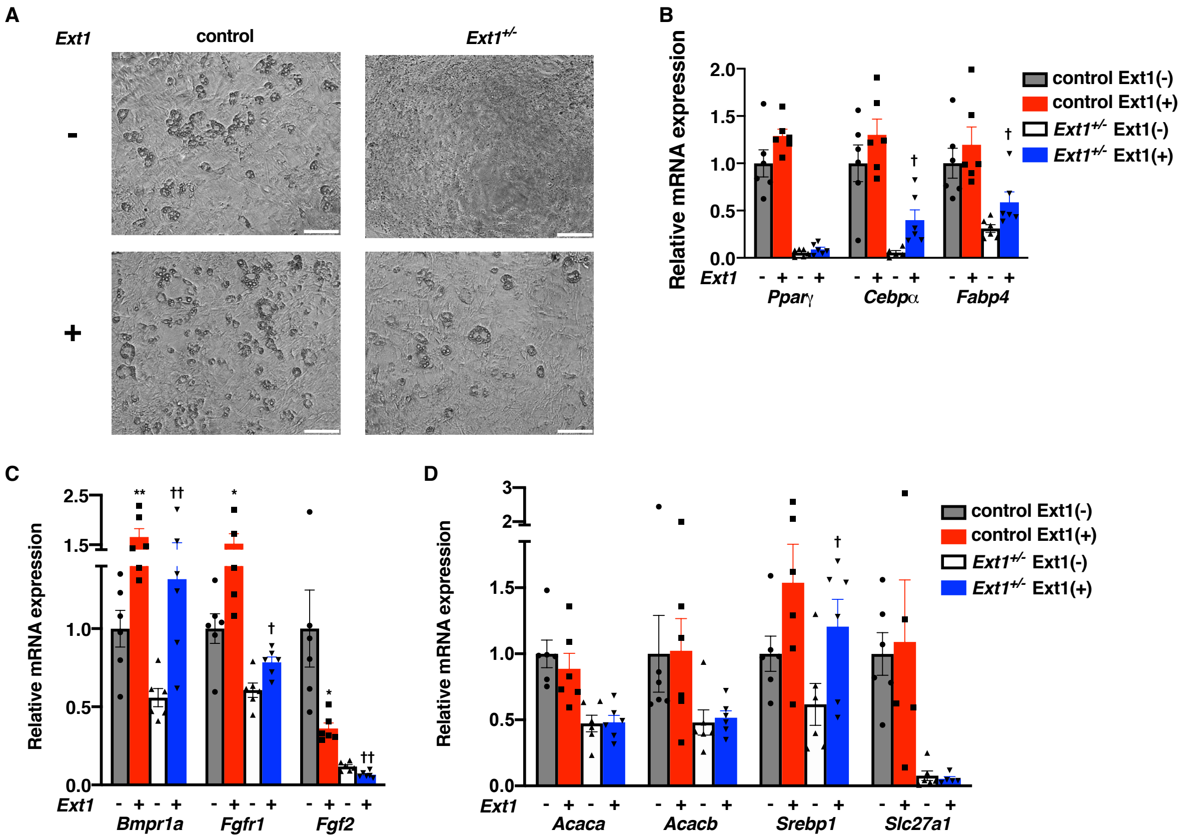

Suppl. Fig. 3. Effect of transient *Ext1* overexpression on 3T3-L1 cells differentiation.

(A) Observation of 3T3-L1 cells at 7 days after inducing differentiation, with or without transient *Ext1* overexpression. Upper pictures: without transient *Ext1* overexpression, lower pictures: with *Ext1* overexpression. White scale bar: 100 μm. (B) Relative mRNA expression levels of differentiation markers at 7 days after inducing differentiation. n = 5 ~ 6. (C) Relative mRNA expression levels of differentiation related genes at 7 days after inducing differentiation. n = 5 ~ 6. (D) Relative mRNA expression levels of adipogenesis related genes at 7 days after inducing differentiation. n = 5 ~ 6. Gray: control cells without transient *Ext1* overexpression at 7 days, red: control cells with transient *Ext1* overexpression at 7 days, white: *Ext1*$^{+/-}$ cells without transient *Ext1* overexpression at 7 days, blue: *Ext1*$^{+/-}$ cells with transient *Ext1* overexpression at 7 days. The mean mRNA expression level of control cells without transient *Ext1* overexpression at 7 day was set to 1. * comparison of gray and red, † comparison of white and blue. *,† $p < 0.05$, **,†† $p < 0.01$.

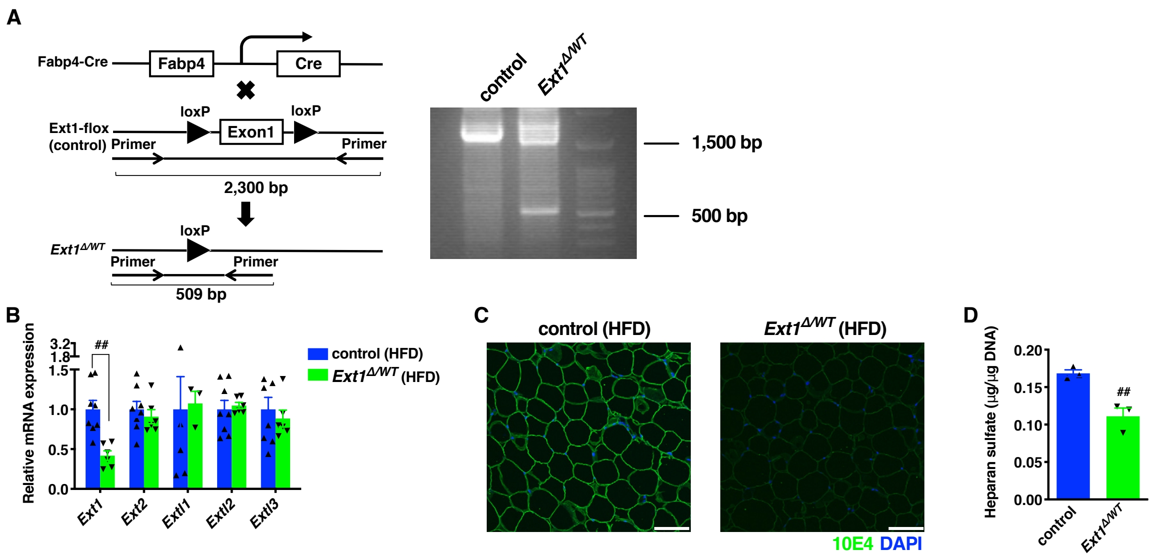

Suppl. Fig. 4. Generation of *Ext1*$^{\Delta/WT}$ *mice*.

(A) Left: schematic overview of the generation of *Ext1*$^{\Delta/WT}$ mouse. Right: DNA extracted from visceral (epididymal) white adipose tissue (vWAT) in control mouse or *Ext1*$^{\Delta/WT}$ mouse was amplified by PCR. Sense primer-GGAGTGTGGATGAGTTGAAG, Antisense primer-CCAAAACTTGGATACGAGCC. (B) Relative mRNA levels of HS synthases in vWAT. n = 3 ~ 8. The mean mRNA expression level of control vWAT was set to 1. (C) Immunohistochemical staining of vWAT in control mouse and *Ext1*$^{\Delta/WT}$ mouse. Green: HS (10E4), Blue: DAPI. White scale bar: 100 μm. (D) Quantification of HS in vWAT. The amount of HS in vWAT was determined by ELISA. n = 3. $^{\#\#}p < 0.01$.

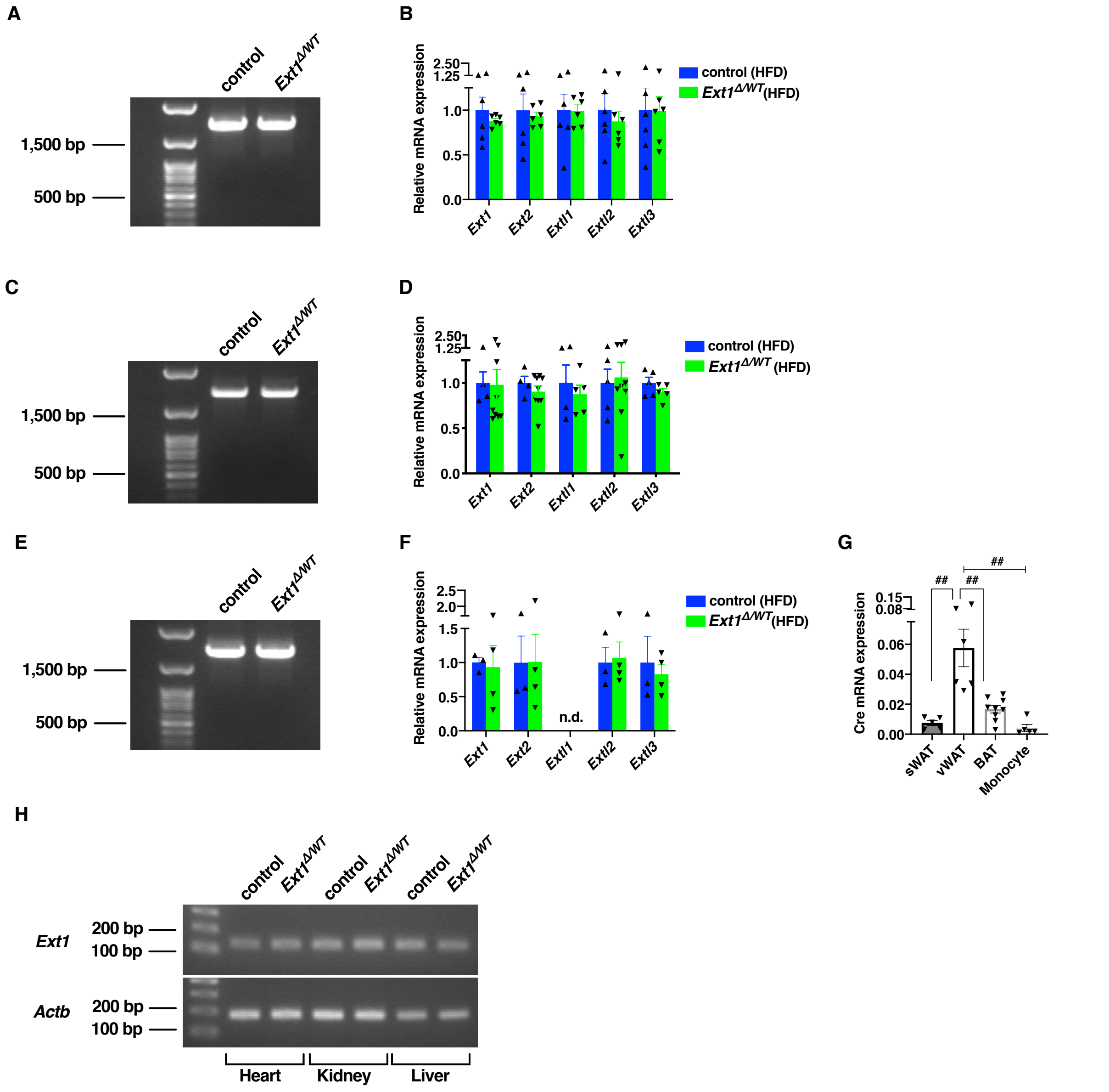

Suppl. Fig. 5. No alteration of *Ext1* in various tissues.

(A) DNA extracted from subcutaneous white adipose tissue (sWAT) in control mouse or *Ext1*$^{Δ/WT}$ mouse was amplified by PCR. (B) Relative mRNA levels of HS synthases in sWAT. n = 6. (C) DNA extracted from brown adipose tissue in control mouse or *Ext1*$^{Δ/WT}$ mouse was amplified by PCR. (D) Relative mRNA expression levels of HS synthases in brown adipose tissue (BAT). n = 4 ~ 9. (E) DNA extracted from monocytes in control mouse or *Ext1*$^{Δ/WT}$ mouse was amplified by PCR. (F) Relative mRNA expression levels of HS synthases in monocytes. n = 3 ~ 4. (G) Comparison of Cre mRNA levels between vWAT, sWAT, BAT, and monocytes. n = 5 ~ 9. (H) mRNA expression levels of *Ext1* in heart, kidney, and liver. In Suppl. Fig. 5B, 5D, and 5F, the mean mRNA expression level of control was set to 1. $^{\#\#}p < 0.01$, n.d.: not detected.

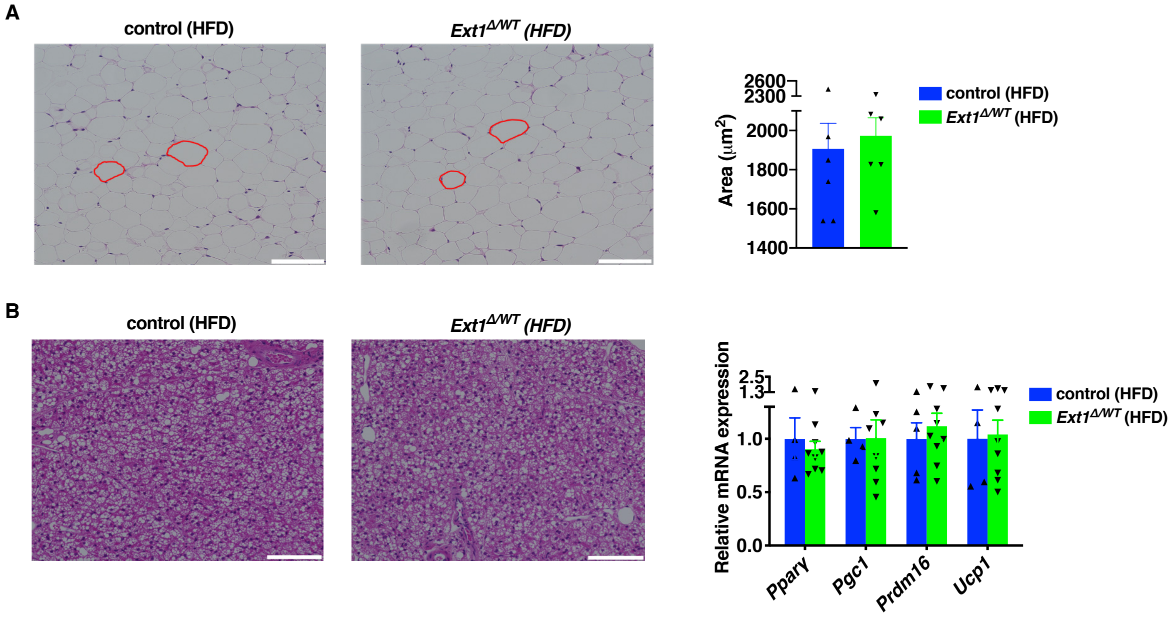

Suppl. Fig. 6. Observation of sWAT and BAT.

(A) Left: Observation of sWAT by HE staining. White scale bar: 100 μm. Right: the averaged area of adipocytes in upper pictures. n = 7. At least 100 areas were measured, per mouse. (B) Left: observation of brown adipose tissue (BAT) in mice fed with HFD by HE staining. White scale bar: 100 μm. Right: relative mRNA expression levels of differentiation markers in the BAT of mice fed with HFD. *Pgc1α*: Pparγ coactivator 1 α, *Prdm16*: PR domain 16, and *Ucp1*: uncoupling protein 1. n = 4 ~ 9. The mean mRNA expression level of control BAT was set to 1. (C) Left: Observation of the liver in mice fed a HFD by Oil Red O staining. Black arrows: lipid area. White scale bar: 100 μm. Right: relative lipid area shown as a percentage relative to the liver area. n = 4 ~ 5.

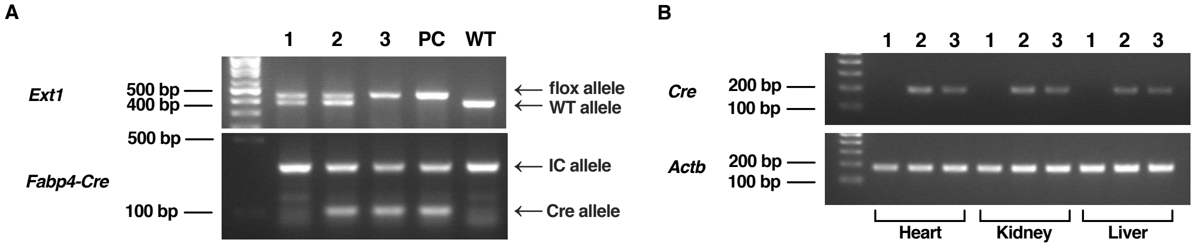

Suppl. Fig. 7. Genotype of mice at embryonic day 16.5 and postnatal day 0.

(A) DNA extracted from tail in mouse at embryonic day 16.5 was amplified by PCR. *Ext1*: Sense primer- GGAGTGTGGATGAGTTGAAG, Antisense primer- CAACACTTTCAGCTCCAGTC. *Fabp4-Cre*: Sense primer (Cre)- GCGGTCTGGCAGTAAAAACTATC, Antisense primer (Cre)- GTGAAACAGCATTGCTGTCACTT, Sense primer (Internal control: IC)- CTAGGCCACAGAATTGAAAGATCT, Antisense primer (Internal control: IC)- GTAGGTGGAAATTCTAGCATCATCC. (B) mRNA expression levels of *Cre* in heart, kidney, and liver at embryonic day 16.5. (A) and (B), 1: $Ext1^{flox/WT}$, 2: $Fabp4\text{-}Cre^{+}$::$Ext1^{flox/WT}$, 3: $Fabp4\text{-}Cre^{+}$::$Ext1^{flox/flox}$, PC: Positive control, and WT: wild type.

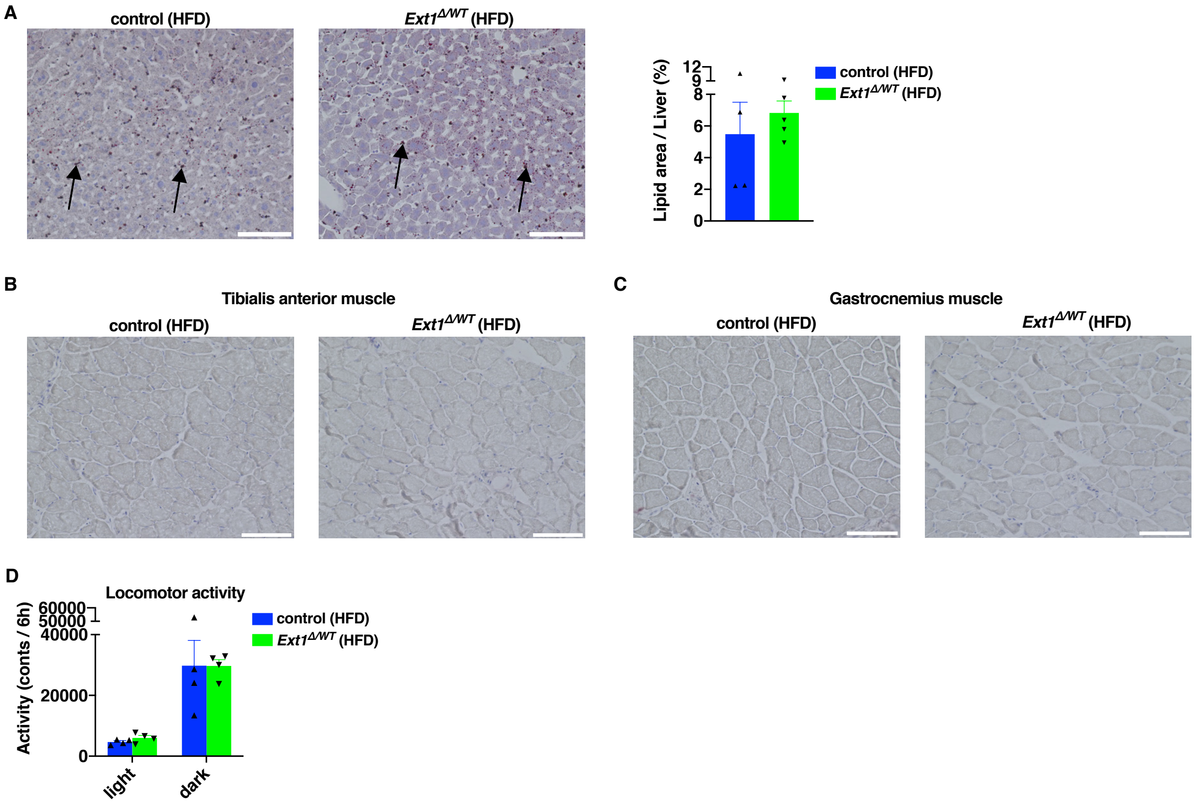

Suppl. Fig. 8. Observation of liver and skeletal muscles.

(A) Left: Observation of the liver in mice fed a HFD by Oil red O staining. Black arrows: lipid area. White scale bar: 100 μm. Right: relative lipid area shown as a percentage relative to the liver area. n = 4 ~ 5. (B) and (C) Observation of the skeletal muscles in mice fed a HFD by Oil red O staining. (B): tibialis anterior muscle, (C): Gastrocnemius muscle. White scale bar: 100 μm. (D) Locomotor activities in the home-cage during the light and dark period. n = 4.

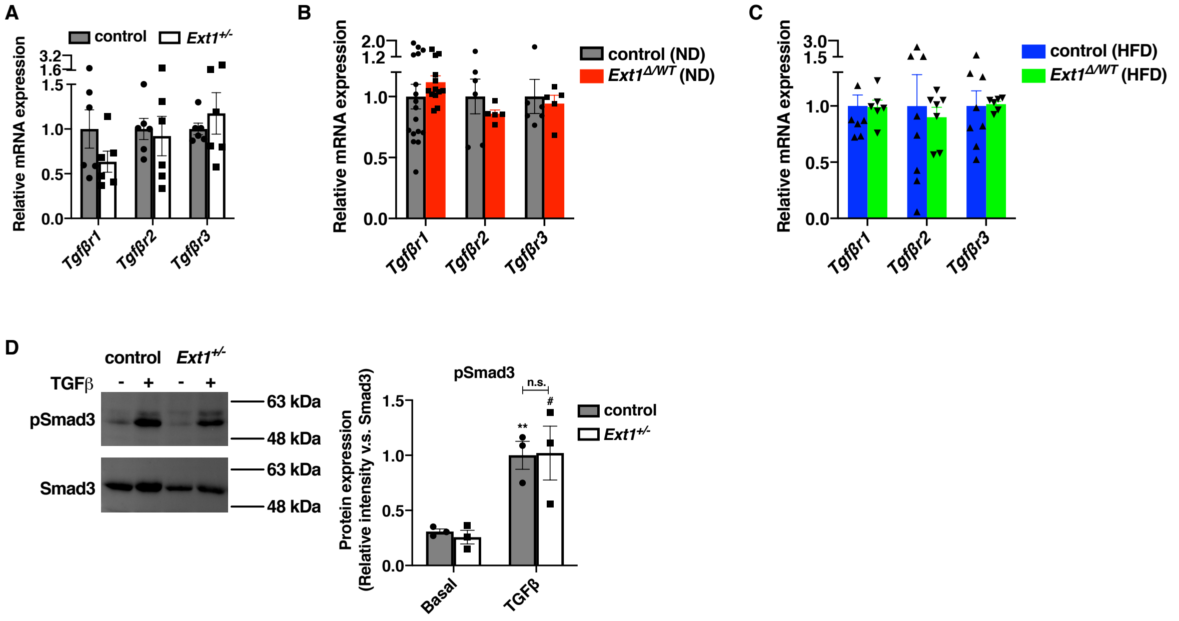

Suppl. Fig. 9. Effect of TGFβ on 3T3-L1 cells differentiation.

(A) Relative mRNA expression of 3T3-L1 cells at 7 days after inducing differentiation in 3T3-L1 cells. n = 6. (B) Relative mRNA expressions of mouse vWAT fed with ND. n = 5 ~ 17. (C) Relative mRNA expressions of mouse vWAT fed with HFD. n = 6 ~ 8. (A), (B), and (C): The mean mRNA expression level in control was set to 1. (D) Left: Representative images of western blot for phosphorylated Smad3 (pSmad) and Smad3 with or without TGFβ treatment. Right: Protein levels of pSmad3 corrected by Smad intensity, with or without TGFβ treatment. n = 3. The mean intensity of control cells with TGFβ treatment was set to 1. * : comparison of control with or without TGFβ treatment. # : comparison of *Ext1*$^{+/-}$ with or without TGFβ treatment. # $p < 0.05$, ** $p < 0.01$.

Suppl. Table 1.

Target sequences for guide RNA in CRISPR-Cas9 system

Position (start-end)		Target sequence (20 mer +PAM, total 23 mer)
#74	74-96	CGGTCGGCAGTGATAGCGCGGGG
#541	541-563	GTCCGCTGTTCGCCCGCCCTGGG
#995	995-1017	CCGGACGCTCTGCGCCCTTTCTT
#1217	1217-1239	AACATTCTAGCGGCCATCGAGGG

Suppl. Table 2.

Primer sequences for quantitative RT-PCR

Target gene		Primer sequence
Acaca	Sense	TGCCTATGAACTCAACAGCG
	Antisense	ACATTCTGTTTAGCGTGGGG
Acacb	Sense	TTTCCGACAAGTGCAGAGTG
	Antisense	CCGCGTTTCCATACTGATCT
Actb	Sense	ACCCGCGAGTACAACCTTCT
	Antisense	CATTCTCCGCAAGATTTGGCA
Bmpr1a	Sense	GCCCTACATCATGGCTGACA
	Antisense	TCCTCCACGATTCCTCCTGT
Bmp4	Sense	ATGGAGCCATTCCGTAGTGC
	Antisense	AGGAATCATGGTGTCTTGACAGA
Cre	Sense	CGACCAGGTTCGTTCACTCA
	Antisense	CAGCGTTTTCGTTCTGCCAA
C/ebpα	Sense	TGAGCCGTGAACTGGACACG
	Antisense	CAGCCTAGAGATCCAGCGAC
C/ebpβ	Sense	TTGATGCAATCCGGATCAAACG
	Antisense	CAGTTACACGTGTGTTGCGTC
C/ebpδ	Sense	AGAACCCGCGGCCTTCTAC
	Antisense	AATGTAGGCGCTGAAGTCGAT
Ext1	Sense	GAAGGAGAAAGCAAGGTTATGAGC
	Antisense	TACGGTGAAGGCAAAATCCAC
Ext2	Sense	CGAGATGAAGGCAAAATCCAC
	Antisense	GTCTCCAGGCATTTTGTAGGTG
Extl1	Sense	GGCACAGGAAGGTCAGCAAC
	Antisense	CACACCACAAAGGCAAAGTCC
Extl2	Sense	CCTGTCATCTTCAAACCACAAAC
	Antisense	TGTCATCGTCTACCATCAACACC
Extl3	Sense	GAACAAGGTAGTGGTGGTGTGG
	Antisense	AATCTCATTCCAGGGCAAGAAC
Fabp4	Sense	TGAAATCACCGCAGACGACA
	Antisense	ACACATTCCACCACCAGCTT
Fgf1	Sense	TTATACGGCTCGCAGACACC
	Antisense	TCTCCGCATGCTTCTTGGAG
Fgf2	Sense	GCTGCTGGCTTCTAAGTGTG
	Antisense	ACTGCCCAGTTCGTTTCAGT

Fgfr1	Sense	GGAGCGCAAGACACAGACAC
	Antisense	AGCACAGCCCAGAAGAGGAG
Glut4	Sense	CTTATTGCAGCGCCTGAGTC
	Antisense	GGGGTTCCCCATCGTCAGA
Ir	Sense	ATGTCCCATCAAATATTGCCAAA
	Antisense	CATCCGGCTGCCTCTTTCT
Irs1	Sense	TTAGGCAGCAATGAGGGCAA
	Antisense	TCTTCATTCTGCTGTGATGTCCA
Klf15	Sense	GCGGAGTCCAGTCACCAC
	Antisense	AACCCACTGAGCATTTCGGT
Pparγ	Sense	GTCTCGGTTGAGGGGACG
	Antisense	GTGTCAACCATGGTAATTTCAGT
Slc27a1	Sense	GCCAGGGATCTCTCTCTCCA
	Antisense	TTGCCTGATCCAGCTTGTCC
Srebp1	Sense	CAGACTCACTGCTGCTGACA
	Antisense	CTCCACTCACCAGGGTCTGC
Tgfβr1	Sense	GATGCTGACTGGGACACCAA
	Antisense	CAAAGTCAAAGCCGCAGGTC
Tgfβr2	Sense	ACGTTCCCAAGTCGGATGTG
	Antisense	TTCAGTGGATGGATGGTCCT
Tgfβr3	Sense	CCTGGTGTGGCATGTGAAGA
	Antisense	AACCCTCCGAAACCAGGAAG

Suppl. Table 3.

Antibodies used in this study

Antibodies	Companies	Dilution	Product Number	Lot
Rabbit monoclonal anti-Actb	Cell Signaling Technology, Danvers, MA, USA	1:1,000	4970S	14
Rabit monoclonal anti-Akt	Cell Signaling Technology,	1:1,000	4691S	11
Rabbit polyclonal anti-Ext1	LifeSpan BioSciences, Seattle, WA, USA	1:1,000	LS-B14297	73858
Rabbit monoclonal anti-Fgfr1	Cell Signaling Technology	1:1,000	9740S	4
Mouse monoclonal anti-heparan sulfate (10E4 epitope)	US Biological, Salem, MA, USA	1:100	H1890	L19031472
Rabbit polyclonal anti-Irs1	Cell Signaling Technology	1:1,000	2382S	4
Rabbit monoclonal anti-pAkt	Cell Signaling Technology	1:2,000	4060S	9
Rabbit polyclonal anti-pIrs1	Cell Signaling Technology	1:500	2381S	5
Rabbit monoclonal anti-Pparγ	Cell Signaling Technology	1:1,000	2443S	4
Rabbit monoclonal anti-pSmad3	Cell Signaling Technology	1:1,000	9520S	16
Rabbit monoclonal anti-Smad3	Cell Signaling Technology	1:1,000	9523S	5
Alexa Fluor 488-conjugated anti-mouse IgM	Abcam, Cambridge, UK	1:1,000	ab15012	GR250433-1

HRP-conjugated anti-rabbit IgG	Jackson Laboratories, West Grove, PA, USA	1:5,000	111-035-144	146133
